# Hypoxia regulates adipose mesenchymal stem cells proliferation, migration, and nucleus pulposus-like differentiation by regulating endoplasmic reticulum stress via the HIF-1α pathway

**DOI:** 10.1186/s13018-023-03818-1

**Published:** 2023-05-08

**Authors:** Jianxin Wu, Lei Yu, Yi Liu, Bing Xiao, Xiaojian Ye, Hong Zhao, Yanhai Xi, Zhicai Shi, Weiheng Wang

**Affiliations:** 1 Department of Orthopaedics, First Affiliated Hospital of Naval Medical University, No. 168 Changhai Road, Shanghai, People’s Republic of China; 2 Department of Orthopedic Surgery and Neurosurgery, No. 906 Hospital of the People’s Liberation Army, Ningbo, Zhejiang People’s Republic of China; 3 Department of Orthopedics, Tianjin First Central Hospital, School of Medicine, Nankai University, No. 24 Kangfu Road, Tianjin, People’s Republic of China; 4 Department of Orthopaedics, Second Affiliated Hospital of Naval Medical University, No. 415 Fengyang Road, Shanghai, People’s Republic of China; 5 Department of Orthopaedics, Tongren Hospital of Shanghai Jiaotong University, No. 1111, Xianxia Road, Shanghai, People’s Republic of China

**Keywords:** Hypoxia, HIF-1α, Migration, Proliferation, ADSCs, Directional differentiation, Endoplasmic reticulum stress

## Abstract

**Objective:**

Hypoxia can promote stem cell proliferation and migration through HIF-1α. Hypoxia can regulate cellular endoplasmic reticulum (ER) stress. Some studies have reported the relationship among hypoxia, HIF-α, and ER stress, however, while little is known about HIF-α and ER stress in ADSCs under hypoxic conditions. The purpose of the study was to investigate the role and relationship of hypoxic conditions, HIF-1α and ER stress in regulating adipose mesenchymal stem cells (ADSCs) proliferation, migration, and NPC-like differentiation.

**Method:**

ADSCs were pretreated with hypoxia, HIF-1α gene transfection, and HIF-1α gene silence. The ADSCs proliferation, migration, and NPC-like differentiation were assessed. The expression of HIF-1α in ADSCs was regulated; then, the changes of ER stress level in ADSCs were observed to investigate the relationship between ER stress and HIF-1α in ADSCs under hypoxic conditions.

**Result:**

The cell proliferation and migration assay results show that hypoxia and HIF-1α overexpression can significantly increase the ADSCs proliferation and migration, while HIF-1α inhibition can significantly decrease the ADSCs proliferation and migration. The HIF-1α and co-cultured with NPCs played an important role in the directional differentiation of ADSCs into NPCs. The hypoxia-regulated ER stress in ADSCs through the HIF-1α pathway, thereby regulating the cellular state of ADSCs, was also observed.

**Conclusion:**

Hypoxia and HIF-1α play important roles in proliferation, migration, and NPC-like differentiation of ADSCs. This study provides preliminary evidence that HIF-1α-regulated ER stress thus affects ADSCs proliferation, migration, and differentiation. Therefore, HIF-1α and ER may serve as key points to improve the efficacy of ADSCs in treating disc degeneration.

## Introduction

Intervertebral disc degeneration (IDD) refers to pathological processes such as nucleus pulposus cell (NPCs) apoptosis, extracellular matrix (ECM) degeneration, inflammation stimulation, and vascular ingrown [1]. The increasing low back pain and neck pain caused by IDD in modern life have become a serious public health problem [2, 3]. At present, the clinical treatments for IDD are mainly conservative management and surgical treatment, neither of which can reverse the IDD process [4].

Transplanting self-renewing stem cells into the degenerative intervertebral discs (IVD) is a hot topic in recent years [5], while the IVD has been shown to be in a physiological hypoxic state and oxygen partial pressure was shown to be decreased from the outer annulus fibrosus (AF) (7.5 kpa) to nucleus pulposus (NP) (0.5 kpa) [6]. Multiple factors in degenerative IVD including hypoxia (1.04–12.67 kPa), inflammation, and oxidative stress may lead to the low survival rate of transplanted stem cells. These greatly restricted the effect of stem cell therapy for IDD [7–9]. It is necessary to optimize stem cell culture conditions for the preservation of cell properties. Studies have shown that hypoxia is a powerful endogenous protective mechanism, which can improve the ability of stem cells to resist injury significantly [10]. The innate ability to survive in a low-oxygen environment is a striking feature of stem cells [11]. Efforts have been made to develop culture conditions similar to the original hypoxic microenvironment of stem cells and find effective culture protocols in vitro ultimately [12]. Since oxygen is a substrate for cellular metabolism, it can even regulate the fate of stem cells directly, as well as their ability to differentiate in multiple directions [13]. Hypoxic-treated stem cells have been used in the treatment of liver and kidney ischemia–reperfusion injury [14, 15], spinal cord injurie [11]. Our previous study showed that hypoxic preconditioning of mesenchymal stem cells (MSC) improved the survival rate of transplanted cells (although the survival rate remains to be improved); thus, improving the effectiveness of stem cell transplantation for IDD is urgent [16]. We also found that hypoxia-inducible factor (HIF) was significantly increased in stem cells pretreated with hypoxic conditions, but the mechanism was not further studied [16]. Therefore, finding a way to keep stem cells active and functional for a long time is the key to solve this problem.

It is proved that the widely expressed HIF-1α is the most important transcription factor that regulates and promotes cell adaptation to hypoxia environment [17]. Many studies have reported that HIF-1α is highly expressed in NP cells (NPCs) and is critical for IDD development and balance [18–20]. Recently, several studies have found that promoting HIF-1α expression in stem cells by gene editing can regulate cell function significantly [21–23]. A series of studies have shown that it is possible to regulate stem cell function to enhance cell-specific functions by editing HIF-1 α genes. There have been no reports that show the enhancement of the efficacy of stem cells for the treatment of IDD by HIF-1 α gene-edited cells.

The endoplasmic reticulum (ER) is a highly conserved organelle responsible for folding and maturing newly synthesized secretory and transmembrane proteins [24]. ER homeostasis may be influenced by various factors, such as high protein requirements, inflammatory processes, reactive oxygen species, or mutated proteins. ER stress, an important mechanism for cell survival, refers to the adaptive response caused by the accumulation of misfolded and unfolded proteins in ER [25]. Oxygen concentration is an important factor in regulating endoplasmic reticulum stress levels [17]. Interestingly, some studies have reported the relationship among hypoxia, HIF-α, and ER stress [26, 27], while little is known about HIF-α and ER stress in ADSCs under hypoxic conditions.

Based on the beneficial effect of hypoxia on stem cells and the special hypoxic microenvironment in IVD as well as on the close relationship between oxygen concentration and ER, a scientific hypothesis was made that hypoxia regulates ER stress in adipose mesenchymal stem cells (ADSCs) through the HIF-1α pathway, thus affecting the biological behavior of ADSCs. Therefore, in this study, the expression of HIF-1α in ADSCs was regulated by gene editing. The effects of HIF-1α on proliferation, apoptosis, migration, and NPC-like differentiation of ADSCs were investigated in vitro. In addition, the changes of ER stress of ADSCs under hypoxic conditions were observed, and their relationships with HIF-1α were preliminarily verified. We expect that this study will provide more effective therapeutic strategies and new targets for the treatment of IDD by stem cell transplantation.

## Methods

### Isolation, identification of ADSCs and NPCs

All SD rats were obtained from the Animal Center of Naval Medical University (Shanghai, China), and all procedures were approved by the Institutional Animal Care and Use Committee of the Naval Medical University. The extraction and identification of ADSCs were performed as reported in our previous study [28]. The third passage (P3) ADSCs were used for subsequent experiments. Similarly, NPCs of SD rats were isolated and digested by type II collagenase at 37 °C for 1 h, and adherent cells were finally obtained.

ADSCs were identified by flow cytometry and three-way induced differentiation. The surface antigen (CD29, CD105, CD45, and CD90) positivity rates of P3 ADSCs were evaluated by flow cytometry (BD, San Jose, CA, USA) to follow the instruction. ADSCs were cultured in induced differentiation medium (Cyagen Biosciences, Santa Clara, CA, USA) according to the instruction. Adipogenesis, differentiation, osteogenic differentiation, and chondrogenic differentiation were verified by Oil Red O staining, alizarin red staining, and Alcian blue staining, respectively. The expression of collagen I and collagen II was determined by immunofluorescence staining to identify the NPCs. Antibodies against collagen I (ab260043, Abcam, Cambridge, MA, USA) and collagen II (ab34712, Abcam, Cambridge, MA, USA) were used. The images were observed and collected under a fluorescence microscope.

P3 ADSCs were planted in culture plates at a density of 1 × 10^5^. When ADSCs were co-cultured with NPCs, the ratio of ADSCs to NPCs was 1:1. For normoxia culture, ADSCs were cultured in complete medium and 21% oxygen concentration at 37 °C for 48 h. ADSCs under hypoxic conditions were cultured in a hypoxic incubator (YCP-30/Q, Huaxi Electronics, Changsha, China) with 1% O_2_, 5% CO_2_, and 94% N_2_ at 37 °C for 48 h. For the detection of ADSCs differentiation, ADSCs were cultured under various conditions for 2 weeks, and the medium was changed every 48 h.

### Overexpression of HIF-1α in ADSCs

P3 ADSCs were digested with 0.25% trypsin and centrifuged, and then, serum-free medium was added to prepare 1 mL single-cell suspension (1 × 10^9^ cells/L). Then, sufficient HIF-1α overexpressing lentiviral particles (about 2 μg) (Funeng, Guangzhou, China) was added to the cell suspension and transfected with a cell electrofusion apparatus (voltage 180 V, pulse width 40 μs). For screening out HIF-1α (+) cell lines with high HIF-1α expression, the change of HIF-1α protein level was determined by Western blot.

### Low expression of HIF-1α in ADSCs

Four target mRNA sequences were designed and synthesized according to the full-length rat HIF-1α gene (no. Nm-024359) provided by GenBank database. The four target mRNA sequences from 5′ to 3′ are UUCAUAAAUUGAACGGCCCTT, AUAAGGGACAAACUCCCUCTT, UUAAGCUUGUCGAAGAGGCTT, and UUUAUCAAGAUGGGAGCUCTT. BLAST search of siRNA in GenBank database showed that the siRNA matched only the rat HIF-1α sequence. Cells were with Lipofectamine 2000 (Invitrogen) according to the manufacturer's instructions. One siRNA without target gene was used as a negative control for RNA interference. The changes of HIF-1α protein level were determined by WB to identify, and the ADSCs lines with low expression of HIF-1α were screened.

### Cell migration assay

Transwell was used to determine the migration of ADSCs. ADSCs were divided into 6 groups according to different pretreated conditions. Normal ADSCs cultured in normoxic (21%) 24 h were NM-NC group; normal ADSCs cultured in hypoxic (1%) 24 h were NM-HC group. ADSCs overexpressing HIF-1αcultured under normoxic conditions (21%) 24 h were the OE-NC group; ADSCs overexpressing HIF-1αcultured under hypoxic conditions (1%) 24 h were the OE-HC group; ADSCs with HIF-1α knock cultured under hypoxic conditions (1%) 24 h were the KD-NC group; and ADSCs with HIF-1α knock down cultured under hypoxic conditions (1%) 24 h were the KD-HC group. The ADSCs concentration was adjusted to 1 × 10^5^ cells/mL, 100 μL of which was inoculated into the upper chamber of Transwell (8 μm, Conring, USA). In addition, 600 μL complete medium was added to the lower chamber and cultured for 6 h or 12 h at 37 °C, washed culture medium with PBS, immobilized the cells with 4% paraformaldehyde (PFA) (Servicebio, Wuhan, China) for 30 min, and then removed the upper layer of unmigrated cells with wet wipes. The remained cells were stained with 0.1% crystal violet for 20 min. Three fields were randomly selected for observation under the microscope. The migration of ADSCs was observed at 6 and 12 h, respectively.

### Cell proliferation assay

Cell proliferations were determined by an EdU assay. ADSCs were divided into 6 groups as above. After 48 h of culture, the EdU detection was performed according to the manufacturer’s instructions (C0078S, beyotime). Before the cells were incubated in a shaker for 10 min, 100 μL 0.5% TritonX-100 was added into each well and then rinsed with PBS. The cells were incubated with Edu solution for 30 min, then washed, and dried subsequently. DAPI staining (Servicebio, Wuhan, China) was used to estimate the total cell number. The slides were sealed and photographed under a fluorescence microscope. Three random fields of each sample were observed and photographed under a fluorescence microscope (Olympus, Japan). The number of positive cells was quantified by the software of ImageJ.

### Western blot

NPCs differentiation and ER stress were measured by WB. Markers of NPCs differentiation included HIF-1α, collage II, aggrecan, and Sox9. ADSCs were grouped according to the HIF expression levels and whether cocultured with NPCs. Transwell co-culture system was used to coculture ADSCs with NPCs. The ADSCs and NPCs concentration was adjusted to 1 × 10^5^ cells/mL, 100 μL of NPCs was inoculated into the upper chamber of Transwell (0.4 μm, Conring, USA), and 100 μL of ADSCs was inoculated into the lower chamber of Transwell. Then the Transwell co-culture system was cultured. ER stress indicators included CHOP, ATF6, and ERK. ADSCs were divided into three groups: normal ADSCs cultured in normoxic conditions (NM-NC), normal ADSCs cultured in hypoxia (NM-HC), and HIF knockout ADSCs cultured in hypoxic conditions (KD-HC).

ADSCs were lysed by RIPA buffer. The proteins were quantified with a BCA protein assay kit (Thermo Fisher Scientific Inc., Waltham, MA), followed by being separated by SDS-PAGE and transferred onto polyvinylidene fluoride (PVDF) membranes. The PVDF membranes were blocked by 5% bovine serum albumin (BSA) and cultured with the primary antibody against the following: HIF-1α (ab8366, Abcam, USA), collagen II (GB11021, Servicebio, Wuhan, China), aggrecan (sc166951, Santa Cruz, Dallas, TX, USA), sox9 (ab185966, Abcam, USA), CHOP(2895,CST), ATF6 (1-40,256, NBP), and ERK (5683p, CST) and β-actin (ab 8226, Abcam, USA). The membrane was washed and incubated with secondary antibodies (Beyotime, Shanghai, China) for 2 h at room temperature; then, ECL kit (Millipore, Bedford, MA, USA) was used to visualize the immunoreactive bands. ImageJ software was used to calculate the band density.

### Statistics analysis

SPSS 20.0 (IBM) and GraphPad 7.0 were used for statistical analysis. Data are presented as the mean ± standard deviation. For normally distributed data, comparisons among groups were made by one-way ANOVA followed by multiple comparisons test (Bonferroni test), and comparisons between two groups were made by *t* test. For non-normal data, the Kruskal‒Wallis test was used for comparison. *P* < 0.05 was considered statistically significant.

## Results

### Identification of NPCs and ADSCs

**Fig. 1:**
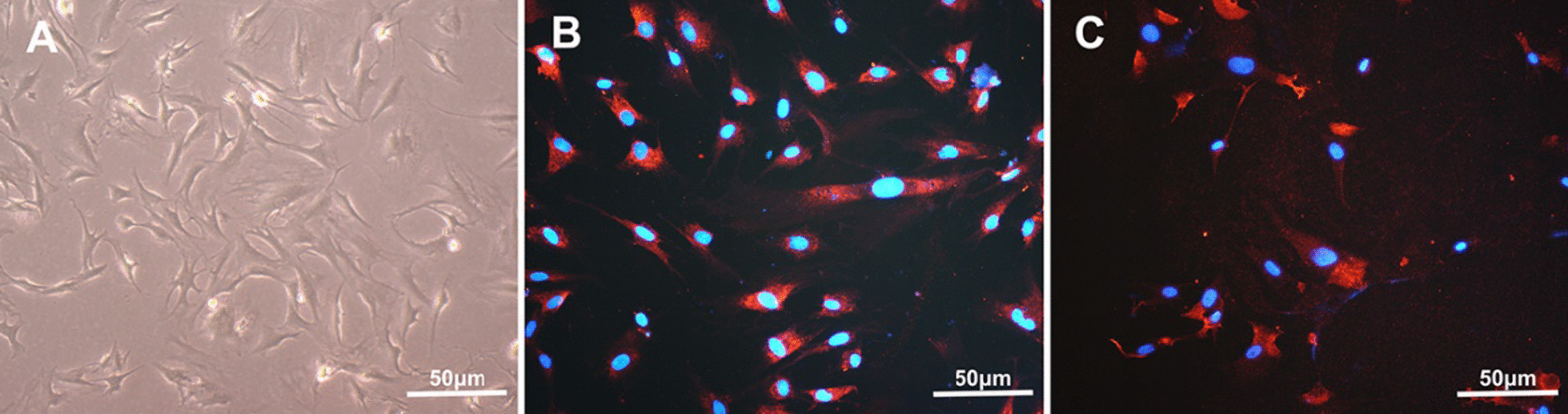
Identification of NPCs. **A** The morphology of NPCs observed under a light microscope; **B** NPCs observed under fluorescence microscope (red staining for COL-2, blue staining for nuclei); **C** NPCs observed under fluorescence microscope (red staining for COL-1, blue staining for nuclei)

**Fig. 2:**
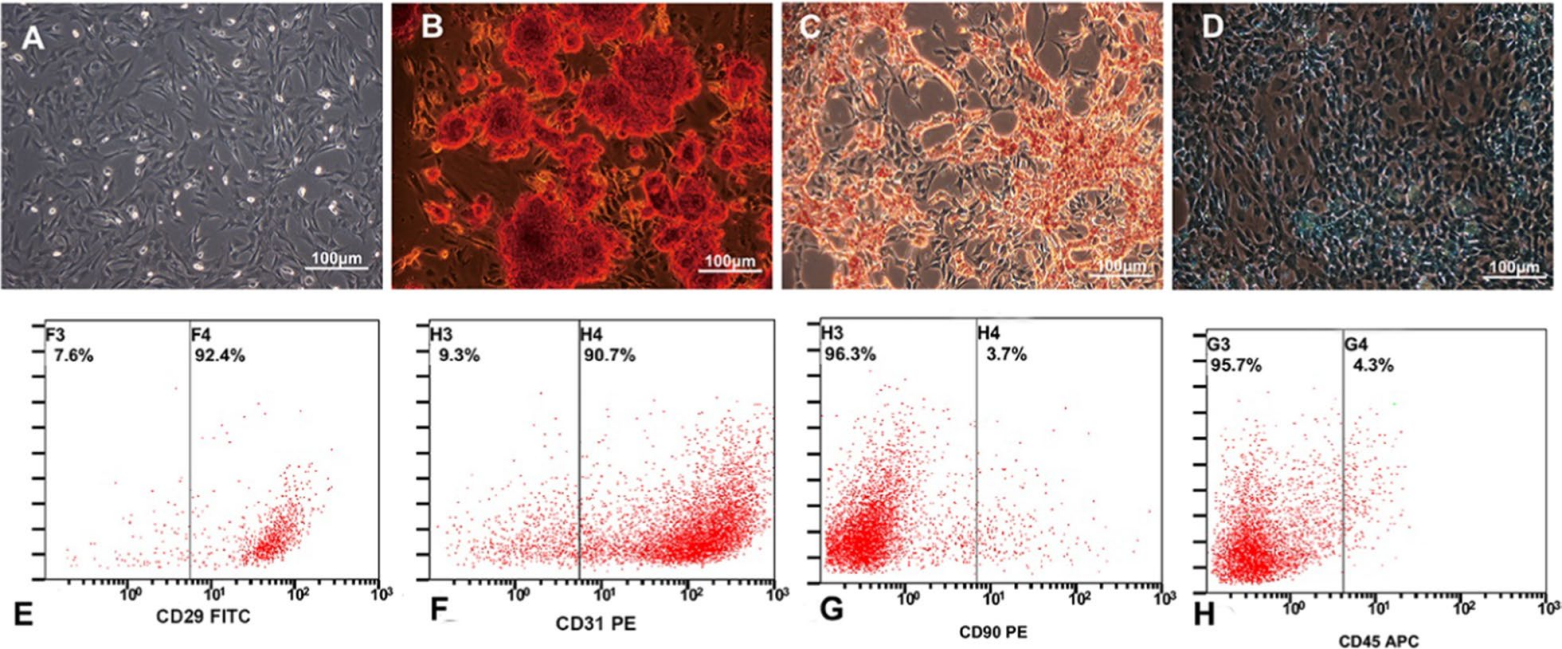
Identification of ADSCs. **A** The morphology of ADSCs observed under a light microscope; **B** the results of alizarin red staining after ADSCs cultured for osteogenic induction. **C** The results of Oil red O staining after ADSCs cultured for adipogenic induction. **D** The results of Alcian blue staining after ADSCs cultured for chondrogenic induction. **E**–**H**: The positive rate of ADSCs antigens CD29 (**E**), CD31 (**F**), CD90 (**G**), and CD45 (**H**) detected by flow cytometry

NPCs were cultured and observed under a light microscope, and evenly distributed adherent spindle-shaped cells can be observed (Fig. 1A). Immunofluorescence staining of NPCs showed that the cells were positive for COL-2 and COL1 (Fig. 1B–C), indicating that the cells were NPCs. ADSCs (P3) were cultured in osteogenic, adipogenic, and chondrogenesis induction conditions for 2–3 weeks. After that, cells were stained with alizarin red, and many red nodules were observed (Fig. 2B). The cells were stained with Oil Red O, and many red-stained lipid droplets were observed (Fig. 2C). Cells were stained with Alcian blue, and a large amount of blue-stained matrix could be observed (Fig. 2D). The results of the three-way induced differentiation test showed that the extracted ADSCs had good multidirectional differentiation potential. The extracted ADSCs were assessed by flow cytometry, and the results of the cell surface antigen assessments showed that the positive rate of CD29 and CD31 was high (> 90%, and that the positivity rate of CD90 and CD45 was low (< 5%) (Fig. 2E–H)). It is proved that most of the obtained cells were ADSCs, which were used in the subsequent experiments.

### The effects of HIF-1α gene expression in ADSCs on cell proliferation

**Fig. 3:**
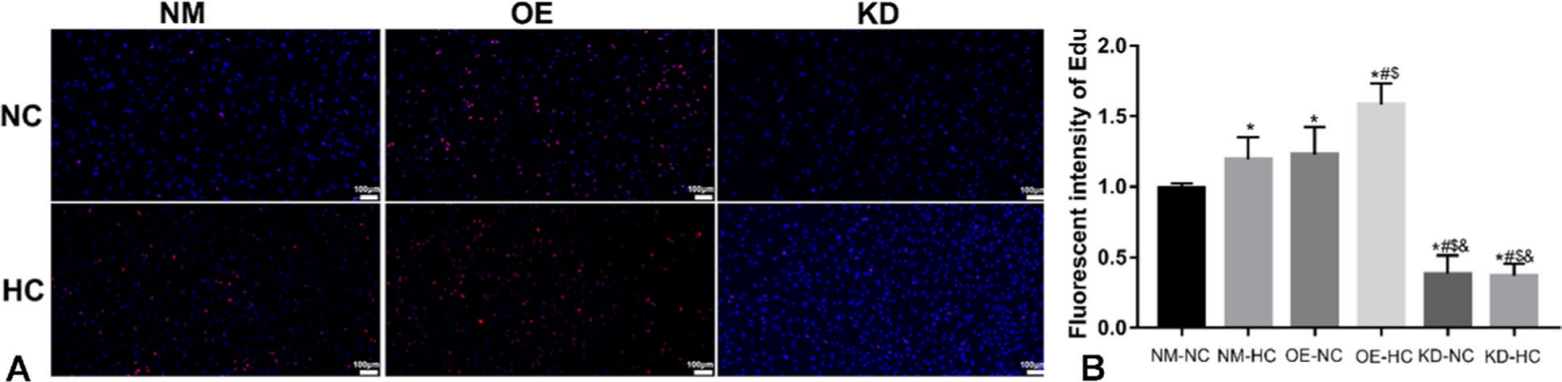
The effects of HIF-1α gene expression in ADSCs on cell proliferation. **A** The red-stained cells were proliferating cells stained with EdU, and the blue fluorescence was stained with nuclei of DAPI. **B** Immunofluorescence cell count in EdU detection. *P < 0.05 versus NM-NC, ^*#*^*P* < 0.05 versus NM-HC, ^$^*P* < 0.05 versus OE-NC, ^&^*P* < 0.05 versus OE-HC (*n* = 5). (*NM* normal, *OE* overexpression, *KD* knock down, *NC* normoxic conditions, *HC* hypoxic conditions)

ADSCs (P3) were transfected with a HIF-1α plasmid, and the changes of HIF-1α protein level in ADSCs after transfection were determined by WB. ADSCs with stable expression (OE) of the HIF-1α protein were used for subsequent experiments. The HIF-1α gene in ADSCs (P3) was silenced by siRNA technology, and a siRNA without any target gene was used as a negative control for the RNA interference. The changes of HIF-1α protein levels were determined by WB. ADSCs with the HIF-1α gene knocked down (KD) were used for subsequent experiments. The results of EdU proliferation assay showed that the ADSC proliferation was significantly increased in 1% hypoxia (NM-HC) for 48 h compared with that in normoxic conditions (NM-NC) (Fig. 2, *P* < 0.05). In normoxic conditions (NC), overexpression of HIF-1α (OE-NC) in ADSCs could significantly increase cell proliferation compared with that in the NM-NC group (Fig. 3, *P* < 0.05), while low expression of HIF-1α in ADSCs (KD-NC) could significantly reduce cell proliferation compared with that in the NM-NC group (Fig. 3, *P* < 0.05). The proliferation of HIF-1α-overexpressing ADSCs (OE-HC) was the greatest (*P* < 0.05) in hypoxic state (HC). The proliferation of ADSCs with low expression of HIF-1α (KD-HC) in hypoxic state (HC) was lower than that of NM-HC group (*P* < 0.05). It is proved that the promoting effect of hypoxia on the proliferation of ADSCs may be through the HIF-1α pathway.

### The effects of HIF-1α gene expression in ADSCs on cell migration

**Fig. 4:**
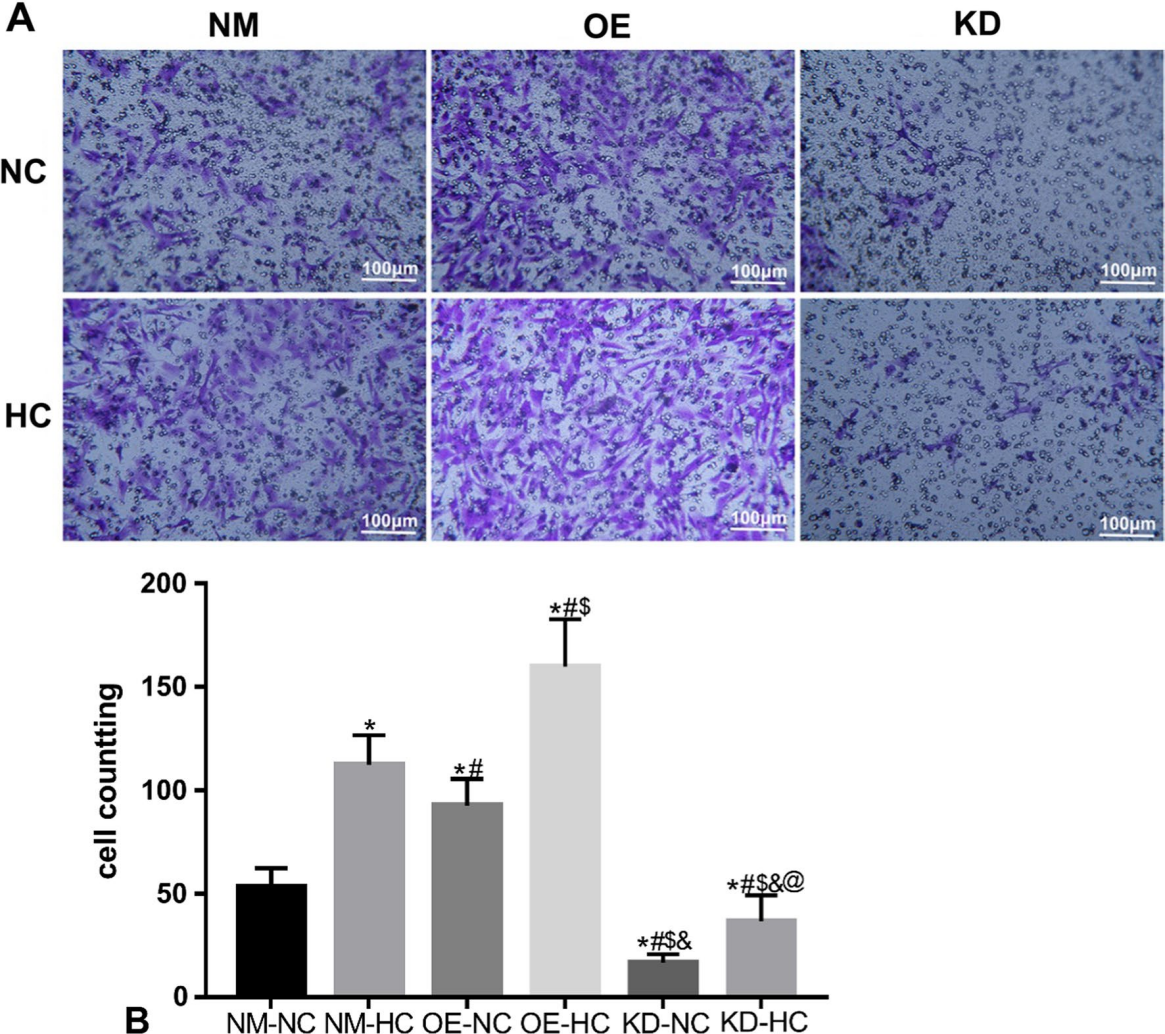
The effects of HIF-1α gene expression in ADSCs on cells migration. **A** Purple represents migrated cells stained with crystal violet. **B** Mean cell counts. *P < 0.05 versus NM-NC, ^*#*^*P* < 0.05 versus NM-HC, ^$^*P* < 0.05 versus OE-NC, ^&^*P* < 0.05 versus OE-HC, ^@^*P* < 0.05 versus KD-HC (*n* = 5). (*NM* normal, *OE* overexpression, *KD* knock down, *NC* normoxic conditions, *HC* hypoxic conditions)

The results of Transwell showed that the migration of ADSCs was significantly increased in 1% hypoxic conditions (NM-HC) for 12 h compared with that in normoxia (NM-NC) (Fig. 4, *P* < 0.05). In normoxic conditions (NC), overexpression of HIF-1α (OE-NC) in ADSCs could significantly increase migration compared with that in the NM-NC group (Fig. 4, *P* < 0. 05), while low expression of HIF-1α in ADSCs (KD-NC) could significantly reduce migration compared with that in the NM-NC group (Fig. 4. *P* < 0.05). The cell migration of HIF-1α-overexpressing ADSCs (OE-HC) was the greatest (*P* < 0.05) in hypoxic state (HC). The cell migration of ADSCs with low expression of HIF-1α (KD-HC) in hypoxic conditions (HC) was lower than that of NM-HC group (*P* < 0.05). It is proved that the promoting effect of hypoxia on the migration of ADSCs may be through the HIF-1α pathway.

### The effects of HIF-1α gene expression in ADSCs on cell directed differentiation into NPC-like cells

**Fig. 5:**
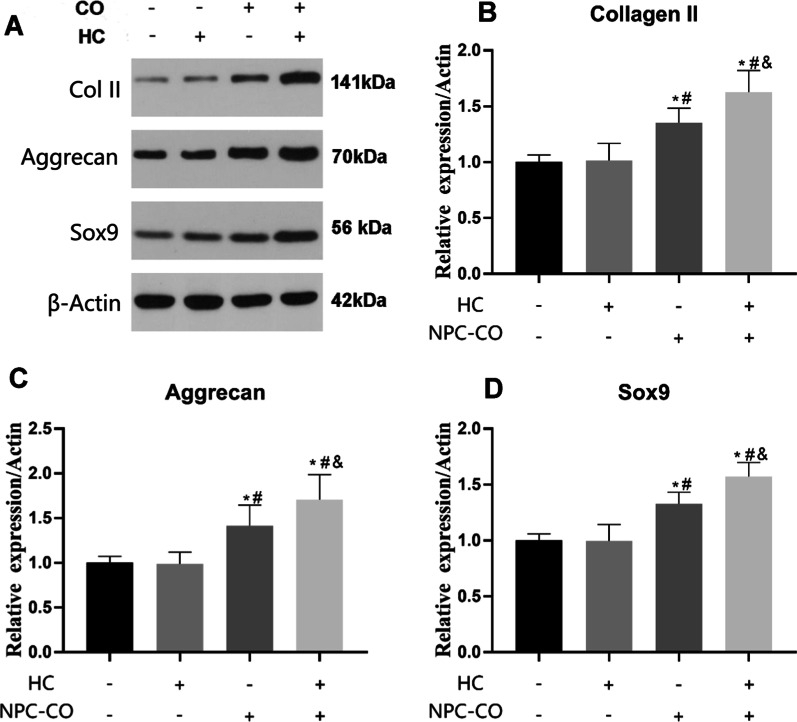
The effects of NPC co-culture and hypoxia on the directed differentiation of ADSCs into NPC-like cells were detected by WB. **A** The protein expressions of Col II, Aggrecan, and Sox9 in ADSCs of each group were detected by WB. **B**–**D** Quantitative counted of  Col II, Aggrecan, and Sox9 in each group. *^#^*P* < 0.05 (*n* = 5). (*CO* co-cultivate, *HC* hypoxic culture)

The effects of different conditions on the directed differentiation of ADSCs into NPC-like cells were determined by WB (Fig. 5A). The results showed that hypoxia could not significantly increase the expression of Collage II (Fig. 5B), Aggrecan (Fig. 5C), and Sox9 (Fig. 5D) proteins in ADSCs compared with the ADSCs under normoxic conditions (*P* > 0.05). In normoxic conditions, coculture of ADSCs and NPCs could significantly increase the Collagen II, Aggrecan, Sox9 protein expression (*P* < 0.05). When ADSCs were co-cultured with NPCs in hypoxic conditions, the differentiation of ADSCs into NPC-like cells was the most obvious (*P* < 0.05, Fig. 5).

**Fig. 6:**
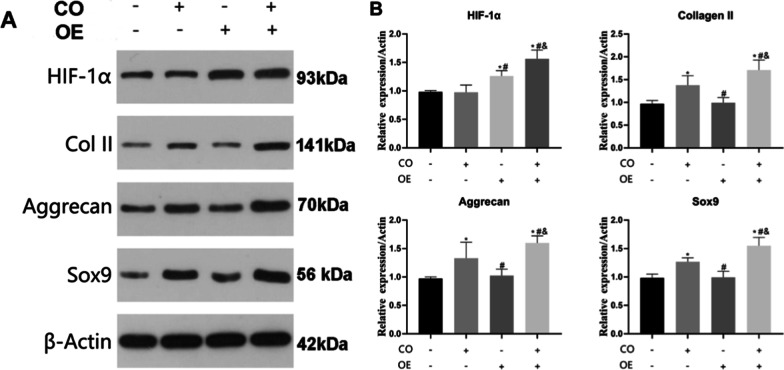
The effects of NPC co-culture and HIF-1α overexpression on the directed differentiation of ADSCs into NPC-like cells were detected by WB. **A** The protein expressions of Col II, Aggrecan, and Sox9 in ADSCs of each group were detected by WB. **B** Quantitative counted in each group. *^#^*P* < 0.05 (*n* = 5)

In ADSCs overexpressing HIF-1α gene, the expression of HIF-1α protein was significantly increased, while the Collage II, Aggrecan, and Sox9 proteins showed no significant change when compared with normal ADSCs (Fig. 6. *P* > 0.05). Co-culture of ADSCs and NPCs could significantly increase the Collage II, Aggrecan, and Sox9 protein expression (Fig. 6. *P* < 0.05). The results of coculture of ADSCs overexpressing HIF-1α gene with NPC showed that the protein expressions of HIF-1α, Col II, Aggrecan, and Sox9 in ADSCs were significantly enhanced (Fig. 6. *P* < 0.05). These results indicated that overexpression of HIF-1α alone could not induce the differentiation of ADSCs into nucleus pulposus cells. The ability of ADSCs to differentiate into NPCs was the greatest under the conditions of co-culture with NPCs and overexpression of HIF-1α gene. These results indicated that co-culture and HIF-1α genes both play an important role in the directional differentiation of ADSCs into NPCs.

**Fig. 7:**
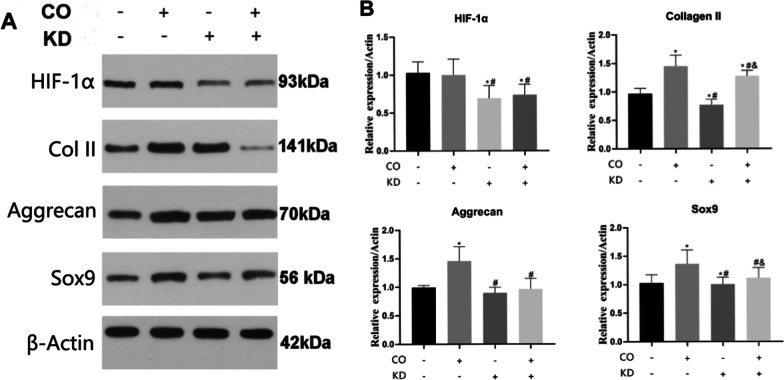
The effects of NPC co-culture and HIF-1α knock down on the directed differentiation of ADSCs into NPC-like cells were detected by WB. **A** The protein expressions of Col II, Aggrecan, and Sox9 in ADSCs of each group were detected by WB. **B** Quantitative counted in each group. *^#^*P* < 0.05 (*n* = 5)

HIF-1α was significantly decreased in ADSCs with low expression of HIF-1α gene compared with other groups (Fig. 7, *P* < 0.05). The low expression of HIF-1α in ADSCs significantly reduced the protein expression of Collage II and Sox9 (*P* < 0.05), indicating that low expression of HIF-1α could inhibit the differentiation of ADSCs into NPCs. The coculture of ADSCs with a low expression of HIF-1α gene and NPCs showed that the protein expressions of HIF-1α, Aggrecan, and Sox9 in ADSCs were not significantly enhanced (*P* > 0.05). These results suggest that both HIF-1α and NPCs coculture have significant roles in regulating the directed differentiation of ADSCs into NPCs. The expression of HIF-1α gene plays an important role in the directional differentiation of ADSCs into NPCs. Inhibiting the expression of HIF-1α gene can effectively inhibit the directional differentiation of ADSCs into NPCs.

### Hypoxia regulates endoplasmic reticulum stress in ADSCs via the HIF-1α pathway

**Fig. 8:**
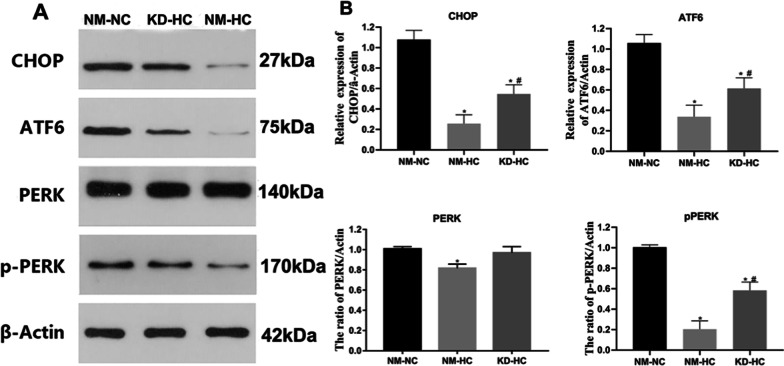
Hypoxia regulates endoplasmic reticulum stress in ADSCs via the HIF-1α pathway. **A** WB detection of CHOP, ATF6, PERK, and p-PERK protein expression in each group. **B** Quantitative counts for each group. *P < 0.05 versus NM-NC, ^#^*P* < 0.05 versus NM-HC (*n* = 5)

Normal ADSCs (P3) and ADSCs with low expression of HIF-1α were cultured in normoxic conditions and 1% hypoxic conditions for 48 h. Detection of ER stress-related markers in ADSCs was carried out by WB. The detection indicators include proteins that respond to endoplasmic reticulum stress: C/EBP homologous protein (CHOP), activating transcription factor 6 (ATF6), and protein kinase R-like endoplasmic reticulum kinase (PERK) and phospho-PERK (p-PERK). The results showed (Fig. 8) that after culturing ADSCs in normoxic conditions for 48 h, the proteins CHOP, ATF6, PERK, and p-PERK that respond to endoplasmic reticulum stress were at higher levels, while the hypoxic culture showed significantly reduced levels of CHOP, ATF6, and p-PERK in the ADSCs. The expression of PERK and p-PERK proteins inhibited endoplasmic reticulum stress (Fig. 8. *P* < 0.05). The endoplasmic reticulum stress level in the KD-HC group was significantly higher than that in the NM-HC group (*P* < 0.05) but significantly lower than that in the NM-NC group (*P* < 0.05). These results suggested that hypoxia regulates ER stress in ADSCs through the HIF-1α pathway, thereby regulating the cellular state of ADSCs.

## Discussion

In this study, we evaluated the effects of hypoxia and HIF-1α on ADSCs and explored possible mechanisms. We found that hypoxia can affect the function of ADSCs (cell proliferation, migration, and directed differentiation into NPCs) through the HIF-1α-mediated endoplasmic reticulum stress pathway. Our study found that the effect of hypoxia on the proliferation, migration, and directed differentiation of ADSCs was mediated by HIF-1α. When the expression of HIF-1α in ADSCs was reduced, the cell proliferation and migration ability were significantly inhibited. However, the directional differentiation of ADSCs into NPCs could not be significantly enhanced when the ADSCs were cultured in a hypoxic environment or by enhancing the expression of HIF-1α in stem cells. The ability of ADSCs to differentiate into NPCs was strongest when HIF-1α and co-cultured with NPCs. This indicates that hypoxia and co-culture system are two key factors that promote the differentiation of stem cells into NPCs. We also found that when hypoxia triggered HIF-1α activation, HIF-1α enhanced the proliferation and migration of ADSCs through the endoplasmic reticulum stress pathway, and directed differentiation toward NPCs.

The key factor affecting the effect of stem cell therapy on IDD is the special microenvironment in the IVD, such as low oxygen, high pressure, and low pH. Among them, the microenvironment of IVD is mainly characterized by hypoxia, which is still the main obstacle to the long-term survival of transplanted mesenchymal stem cells. The hypoxic environment plays a crucial role in the progression of IDD [29]. Under physiological conditions, the oxygen partial pressure in the intervertebral disc decreased sharply from the outer annulus fibrosus (7.5 kPa) to the nucleus pulposus (0.5 kPa) [6]. During IDD, the oxygen partial pressure in the nucleus pulposus increases (1.04–12.67 kPa) due to the ingrowth of blood vessels and the rupture of the annulus fibrosus [7]. During IDD, the water in NP tissues is reduced, and the diffusion of oxygen, nutrients, and metabolic wastes is restricted. In the IVD microenvironment, oxygen tension is low due to poor diffusion, followed by increased lactate production through anaerobic metabolism [30]. Lactic acid excretion is blocked and gradually accumulates, which leads to a decrease in pH, which affects cellular metabolism and function [30]. With the progression of IDD, the microenvironment within the IVD deteriorates, which leads to a significant decrease in the efficacy of cell transplantation in the treatment of IDD. Our study found that hypoxic preconditioning of mesenchymal stem cells improved the survival rate of transplanted stem cells. We also found that HIF was significantly increased in stem cells pretreated with hypoxic conditions [16]. However, the hypoxic environment in vitro (1%) cannot fully simulate the hypoxic environment in IVD. Stem cells need to undergo a multistep process in a normoxic environment before transplantation into IVD, which leads to changes in stem cell status and function. In addition, the complex microenvironment in IVD may affect the long-term maintenance of specific functions of stem cells after hypoxic preconditioning. Therefore, finding a way to keep stem cells active and functional in hypoxic conditions for a long time is the key to solving this problem. This article provided a theoretical basis for gene-edited stem cell transplantation for the treatment of IDD, by studying the effect of hypoxia-critical HIF-1α gene expression in ADSCs on stem cell proliferation, migration, and directional differentiation into NPCs.

Hypoxia can activate the expression of HIF-1α, which is widely expressed in almost all cells in the human body. Benita et al. [31] proposed that HIF 1α has the potential to regulate 81 genes under hypoxic conditions in multiple cell types. It also plays an important role in the occurrence and progression of many diseases, including myocardial ischemia [32, 33], tumors [32, 34], and chronic degenerative diseases [35–40]. HIF has a unique regulatory role in the function of NPCs and is closely related to the occurrence and progression of IDD [41]. The HIF pathway can regulate various functions such as cell proliferation, cell metabolism, glycolysis, mitochondrial electron transport chain (ETC), lactate/H + efflux, which plays an important role in the normal metabolism and degeneration of IVD. Hypoxia significantly increases the phenotype of NPCs with little effect on the annulus fibrosus, suggesting that HIF-1α may serve as a marker for NPCs [36, 42]. However, the findings regarding the regulation of ECM metabolism by HIF 1α remain controversial. A growing number of studies have shown that hypoxia induces upregulation of HIF-1α and promotes ECM synthesis, including NPCs [36, 42, 43], chondrocytes [44, 45], fibroblasts [46], and mesenchymal stem cells [45]. Animal and human studies have shown that the content of ECM synthesis is inversely correlated with the degree of hypoxia [36, 42, 47, 48]. The ideal hypoxia level to promote NPC survival is 1% of the physiological oxygen concentration [49], and therefore 1% oxygen concentration was used as the hypoxic culture condition in this experiment. The synthesis of glycosaminoglycans (GAGs) in NPCs has been shown to increase significantly with the gradual decrease of oxygen content in the medium. However, at 2% oxygen, no increase in ECM synthesis was observed despite HIF-1α activation [48]. These suggest that HIF-1α is not the only factor promoting ECM synthesis. Factors affecting the progression of IDD may also include age, gender, histological origin, and severity of injury model [48]. HIF-1α plays a complex and important role in IVD metabolism. Therefore, regulating the functional status of the intervertebral disc through the HIF-1α pathway is a promising direction for the treatment of IDD, but its specific mechanism remains to be further studied. Exploring and analyzing the pathway of HIF-1α promoting or inhibiting ECM synthesis in NPCs, and finding out its most critical regulatory mechanism will be the focus of future research.

Hypoxia can modulate stem cell plasticity through the action of HIF. The stable and high expression of HIF in stem cells can stably regulate the state and function of stem cells. HIF-1α regulates cellular metabolism through transcriptional activation of genes that regulate glycolysis, such as GLUT1 and PDK1, and anaerobic metabolism [50, 51]. Oxygen concentration maintains stem cell function by regulating HIF-1α and energy metabolism [52]. HIF also affects the immunomodulatory properties of stem cells. Silencing of HIF-1α in MSCs reduces inflammation and inhibits pro-inflammatory T cell generation [53]. HIF can reduce apoptosis by downregulating p53 [54]. p53 can induce the transcriptional activation of p21 and participate in the regulation of apoptosis and cell cycle [55]. The ability of HIF to significantly enhance stem cell function has been demonstrated in ADSCs [56], foreskin mesenchymal cells [57], and bone marrow mesenchymal cells [57]. Under hypoxic conditions, HIF-1α stabilizes and accumulates in the nucleus and triggers transcriptional activation [58]. We demonstrated a significant increase in cell proliferation and migration in hypoxic conditions. We also found that upregulation of HIF-1α can also increase the proliferation and migration of ADSCs and that downregulation of HIF-1α can reduce the proliferation and migration of ADSCs. This indicated that hypoxia increased the proliferation and migration of ADSCs through HIF-1α-dependent transcriptional activity. In addition, HIF-1α upregulation can significantly increase the directed differentiation ability of ADSCs into NPCs. Previous studies have shown that hypoxic preconditioning significantly enhances mesenchymal stem cell extracellular matrix synthesis and migration, both of which are dependent on HIF-1α [16, 59]. In addition, in severely degenerative IVD, neovascularization is accompanied by an increase in oxygen concentration and a decrease in pH, which may impair the efficacy of stem cell therapy [60]. However, whether the transplantation of exogenous HIF-1α-overexpressing ADSCs can improve its therapeutic effect on IDD remains to be further investigated.

The endoplasmic reticulum stress level in ADSCs under hypoxic culture conditions was significantly decreased, and the expression of HIF-α was significantly increased. The endoplasmic reticulum (ER) is a highly conserved organelle responsible for the folding and maturation of newly synthesized secreted and transmembrane proteins. ER homeostasis may be affected by different stimuli, such as high protein demand, inflammatory processes, reactive oxygen species, or mutated proteins, leading to the accumulation of misfolded and unfolded proteins in the ER lumen. This condition is known as ER stress and results in an adaptive response known as the unfolded protein response, which is known to be an important mechanism for cell survival [25]. Oxygen concentration is an important factor in the regulation of ER stress levels. HIF-1α plays a central role in the hypoxia signaling pathway [17]. The regulation of the self-renewal properties of stem cells by oxygen concentration is also indirectly dependent on the stabilization of HIF-1α. HIF-1α can directly regulate the activation of stem cell genes (such as Oct-4, Sox-2, or Nanog) and is a key determinant of factors affecting cell metabolism [61]. The interrelationship of hypoxia, HIF-1α, and ER stress has been reported in other cell types [26, 27].Our findings further confirmed that hypoxia regulates endoplasmic reticulum stress in ADSCs via the HIF-1α pathway, thereby regulating the cellular state of ADSCs. The specific mechanism and in vivo application of ER stress in regulating the proliferation, migration, and differentiation of ADSCs into NPCs through HIF-1α pathway remains to be further being verified and studied.

There were some limitations in this study. First, this study was only in vitro cell tests. We found that hypoxia can regulate ADSCs proliferation, migration, and nucleus pulposus-like differentiation by regulating endoplasmic reticulum stress via the HIF-1α pathway at the cellular level. The molecular mechanism needs further confirmation in vivo animal experiments. Secondly, the direct relationship between ER stress and ADSCs proliferation, migration, and differentiation, and the target genes up-regulated by HIF-1α/ER stress in ADSCs need to be further studied. In the future, preclinical trials and animal studies should be using ADSCs overexpressing HIF-1α for intervertebral disc injection, which may provide a therapeutic strategy for optimizing stem cell therapy for IDD.

## Conclusion

This study confirmed that hypoxia promotes ADSC proliferation, migration, and directed differentiation into NPCs, by activating the HIF-1α-mediated endoplasmic reticulum stress signaling pathway. These reveal the adaptation of ADSCs to hypoxia in the degenerative disc microenvironment mechanism. However, the target genes upregulated by HIF-1α/ER stress remain to be further investigated. Further studies should investigate the long-term survival effects of transplanted ADSCs overexpressing HIF-1α or ER stress in animal models of IDD. Preclinical and animal experiments using HIF-1α-overexpressing ADSCs for intradiscal injection may provide a therapeutic strategy for optimizing stem cell therapy in IDD therapy.

## Data Availability

All data relevant to the study are included in the article. Please contact the corresponding author if additional information is required.

## Abbreviations

ER: Endoplasmic reticulum
NPCs: Nucleus pulposus cells
ADSCs: Adipose mesenchymal stem cells
IDD: Intervertebral disc degeneration
ECM: Extracellular matrix
IVD: Degenerative intervertebral discs
AF: Annulus fibrosus
NP: Nucleus pulposus
MSC: Mesenchymal stem cells
HIF: Hypoxia-inducible factor
PVDF: Polyvinylidene fluoride
NM: Normal
OE: Overexpression
KD: Knock down
NC: Normoxic conditions
HC: Hypoxic conditions
